# Toxic Effects of the Most Common Components of Energetic Co-Crystals

**DOI:** 10.3390/molecules30153234

**Published:** 2025-08-01

**Authors:** Xinying Peng, Cunzhi Li, Huan Li, Hui Deng, Xiaoqiang Lv, Ting Gao, Jiachen Shen, Bin Zhao, Zhiyong Liu, Junhong Gao

**Affiliations:** 1 Toxicology Research Center, Institute for Hygiene of Ordnance Industry, NO. 12 Zhangbadong Road, Yanta District, Xi’an 710065, China; 2 Xi’an Key Laboratory of Toxicology and Biological Effects, NO. 12 Zhangbadong Road, Yanta District, Xi’an 710065, China

**Keywords:** Cocrystal Materials, CL-20, Toxicities, Toxic Mechanism, Energetic Materials

## Abstract

Hexanitrohexaazaisowurtzitane (CL-20) is a high-energy-density material known for its exceptional explosive performance, but it suffers from significant safety concerns due to its high sensitivity. To mitigate this issue, researchers have explored the synthesis of CL-20-based cocrystals with other energetic materials to achieve a balance between energy output and safety. Recent advancements in CL-20 cocrystals have focused on developing novel synthesis methods and leveraging computational design techniques to predict and optimize their physicochemical properties. However, the toxicity of CL-20 cocrystals, along with their environmental and health risks, remains a critical concern. This review systematically examines recent progress in CL-20 cocrystal energetic materials, emphasizing toxicity profiles and mechanistic insights into their components. The findings serve as a foundation for the development of safer energetic materials, thereby facilitating sustainable advancements in manufacturing technologies and industrial applications of CL-20.

## 1. Introduction

**Figure 1:**
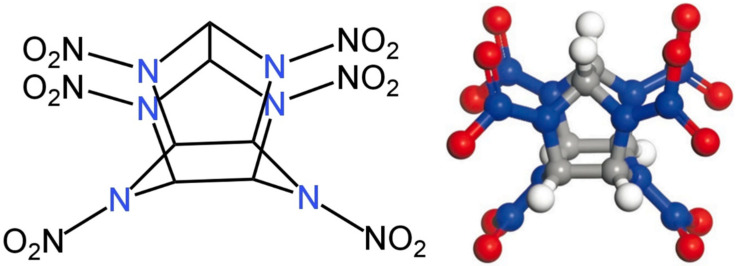
The chemical structure (**left**) and ball-and-stick model (**right**) of CL-20. The gray ball in ball-and-stick model is Carbon (C), blue is Nitrogen (N), red is Oxygen (O) and white is Hydrogen (H).

High-energy-density materials (Energetic Materials, HEDMs, EMs), including explosives, gunpowder, and propellants, have been extensively utilized in military and civilian applications, playing a pivotal role in aerospace, defense, and industrial production [1]. Among these, 2,4,6,8,10,12-hexanitro-2,4,6,8,10,12-hexaazaisowurtzitane (Hexanitrohexaazaisowurtzitane, HNIW, CL-20) represents one of the most advanced energetic materials due to its superior performance characteristics. CL-20 belongs to the class of cage-like nitramine explosives, characterized by complex polycyclic structures that enhance their energetic properties (Figure 1). Since its synthesis in 1987, CL-20 has attracted considerable research attention [2].

Compared to conventional energetic materials such as octahydro-1,3,5,7-tetranitro-1,3,5,7-tetrazocine (HMX), triaminotrinitrobenzene (TATB), cyclotrimethylene trinitramine (RDX), and 2,4,6-trinitrotoluene (TNT), CL-20 exhibits superior oxygen balance (OB, −10.95%), higher density (>2 g/cm^3^), and enhanced detonation performance (>9000 m/s) [3]. Furthermore, it demonstrates favorable thermal stability, making it a promising candidate for applications in propellants, gunpowder, and rocket fuels [4]. Consequently, many developed nations, particularly in Europe and North America, have identified CL-20 as a potential alternative to RDX and HMX, prompting extensive research into its synthesis and applications.

However, despite its outstanding energetic properties, CL-20 exhibits high mechanical sensitivity, leading to significant safety challenges in processing, handling, and transportation. These limitations have severely restricted its practical applications [5]. Addressing the trade-off between energy density and safety, or developing energetic materials that simultaneously exhibit high energy and low mechanical sensitivity, has remained a central challenge in energetic material research. Various strategies have been explored to mitigate this issue, including the incorporation of polymer binders in polymer-bonded explosives and the development of novel low-sensitivity energetic materials [6].

Among these approaches, cocrystallization has emerged as a promising and effective strategy for enhancing the safety of energetic materials without compromising their energetic performance [7]. Cocrystal technology enables the combination of different high-energy molecules within a single crystalline lattice, forming multi-component molecular crystals known as cocrystals. These structures are stabilized through intermolecular interactions such as hydrogen bonding, π–π stacking, van der Waals forces, and halogen bonding. These intermolecular forces play a crucial role in stabilizing the crystal lattice and mitigating issues associated with polymorphism in single -component energetic materials [8]. Cocrystallization can significantly alter the physicochemical properties of energetic materials, including density, melting point, and mechanical sensitivity, without modifying the fundamental molecular structure of the original energetic compound. This technique provides an effective means of optimizing the balance between energy output and safety, allowing the resulting energetic materials to retain high detonation performance while exhibiting reduced impact sensitivity [9]. For instance, due to the stronger hydrogen bonding in the cocrystal and the reduced reactivity of CL-20 induced by 1-methyl-dinitroimidazole based crystals (4,5-MDNI), the CL-20/4,5-MDNI cocrystal exhibits low impact sensitivity (impact sensitivity: 11 J) while maintaining outstanding detonation performance (detonation velocity: 8915 m·s^−1^, detonation pressure: 35.88 GPa) and thermal stability (223 °C) [3,10]. Through the strategic selection of coformers, cocrystallization has been shown to improve thermal stability, detonation characteristics, mechanical resilience, and overall safety profiles of energetic materials [11]. Consequently, the design, synthesis, and application of cocrystal energetic materials have become a highly active area of research in recent years.

Despite these advancements, existing research on CL-20 cocrystals has predominantly focused on their synthesis, structural characterization, interaction mechanisms, and computational modeling. Most experimental studies on cocrystal materials remain at the laboratory scale, and investigations into their toxicity, environmental impact, and biological effects are still in their infancy. There is a significant gap in understanding the toxicities, toxic mechanisms of CL-20 cocrystals, and the potential health risks associated with their components.

This review aims to systematically examine the development of CL-20 cocrystals, with a particular emphasis on their toxicity profiles and toxicological mechanisms. By integrating findings from existing studies and available data, this work seeks to identify key factors influencing the toxicity of CL-20 cocrystals and propose potential directions for future research. The insights provided in this review are expected to contribute to a deeper understanding of the safety and environmental considerations of CL-20-based energetic materials, ultimately guiding the development of safer and more sustainable alternatives for industrial and military applications.

## 2. Advances in Cocrystal Materials Research

**Figure 2:**
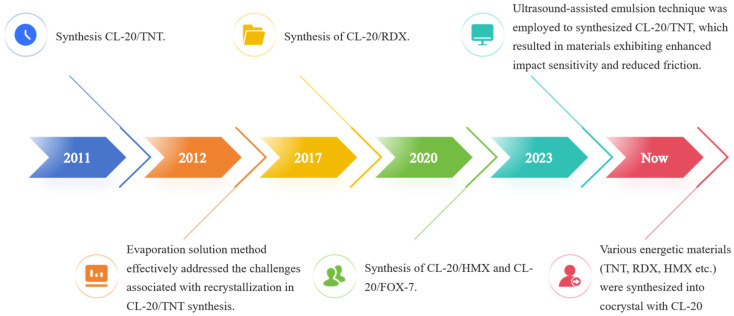
Advances in synthesis of CL-20 cocrystal materials [13,14,15,16,17,18,19,20,21,22,23].

In the year 2011, Bolton and his associates achieved the successful synthesis of a cocrystal energetic material comprising a 1:1 molar ratio of CL-20 and TNT. This significant advancement has paved the way for further exploration in the field of energetic materials and has generated heightened interest among researchers regarding cocrystal energetic materials [12]. To date, numerous energetic materials (TNT, RDX, HMX, 1,1-diamino-2,2-dinitroethylene (FOX-7), glyceryl trinitrate (NQ), 1H-1,2,4-Triazol-3-amine,5-nitro-(9CI) (ANTA), 1-Methyl-2,4-dinitroimidazole(2,4-MDNI), 4,5-MDNI, and N,N-dimethylformamide (DMF), etc.) have been employed in the synthesis of CL-20 cocrystal (Figure 2) [13,14,15,16,17,18,19,20,21,22,23]. CL-20 is synthesized into a composite material through the cocrystallization method with other energetic substances, effectively addressing the inherent conflict between energy output and safety associated with conventional elemental energetic materials. This approach establishes a robust foundation for large-scale production and facilitates comprehensive research into various attributes, including crystallization mechanisms, detonation characteristics, and safety performance [24]. The modification of energetic materials via cocrystallization has emerged as a potent approach for the development of novel energetic compounds and the enhancement of their properties.

In the course of synthesizing diverse CL-20 cocrystal energetic materials, the structural mechanisms underlying these cocrystal formations are persistently investigated [25]. In recent years, there has been a notable increase in scholarly investigations focusing on the swift and efficient implementation of computational design methodologies for energetic materials [26]. The system is capable of providing a detailed visualization of the microstructure of energetic materials at the molecular level, as well as calculating a range of physical and chemical properties associated with these materials [27]. Additionally, it serves as a substitute for conventional chemical synthesis, structural analysis, and physical monitoring experiments, which contributes to a reduction in development time, lowers associated costs, and mitigates certain risks inherent in the experimental process [28]. Currently, the integration of experimental testing with theoretical calculation methodologies has emerged as the predominant approach for investigating the interactions among eutectic components [29]. The findings revealed that, in addition to the C–H hydrogen bonds formed between the nitro oxygen of CL-20 and the aliphatic hydrogen of TNT, there occur non-covalent van der Waals interactions within the cocrystal [14]. These interactions include the engagement between the aromatic electron-deficient ring of TNT and the nitro group of CL-20, as well as the nitro–nitro interactions between the two constituent components. However, due to the absence of strong, predictable interactions in their chemical structures, preparing cocrystals and studying their formation mechanisms are generally challenging in practice [14].

However, to date, no systematic research on cocrystal material toxicity has been identified. As novel high-energy materials, cocrystals warrant critical toxicity research for national security and environmental health. Cocrystal materials may pose risks to human health through inhalation or dermal contact during production, storage, and usage. Their decomposition products might exhibit carcinogenicity, c, or bioaccumulate potential, with long-term exposure leading to hematopoietic system damage and neurological disorders. Such studies of their toxicities would not only refine safety protocols for energetic material applications but also guide green cocrystal engineering—optimizing molecular structures of co-formers to reduce ecotoxicity while preserving energy performance. It indicates that the mechanistic study of cocrystal material toxicities will emerge as a highly promising area within the field of materials science.

## 3. Toxic Effects of Common Components in CL-20 Cocrystal Materials

### 3.1. CL-20

CL-20 exhibits slight toxicity in Sprague-Dawley rats (LD_50_ is 5000 mg/kg) [30]. It has weak skin sensitization potential in guinea pigs, minimal skin irritation in rabbits, and sub-chronic oral toxicity in rats [30,31,32,33,34,35]. Genotoxicity assessments using the alkaline comet assay indicate that CL-20 induces DNA strand breaks in hamster lung cells (V79), confirming its potential genetic toxicity [36]. Acute oral toxicity studies in Japanese quail reveal that exposure to CL-20 results in a dose-dependent decrease in body weight gain as well as increased liver weight, plasma sodium levels, and creatinine. Furthermore, reproductive toxicity studies indicate that exposure to CL-20 leads to significant, dose-dependent reductions in embryo weight, with multiple craniofacial malformations, including skull deformities and beak curvature [37].

The persistence of CL-20 in the environment is largely attributed to its multiple electron-withdrawing N–NO_2_ functional groups, which hinder its degradation by oxidative enzymes under aerobic soil conditions. As a result, CL-20 undergoes slow and incomplete degradation, leading to long-term contamination of soil and water bodies [38]. CL-20 has been reported to cause both acute and chronic toxicity in soil invertebrates such as earthworms and nematodes, including reversible neurotoxicity and reproductive toxicity in earthworms [39,40]. Compared to other energetic materials such as RDX, HMX, and TNT, CL-20 exhibits higher toxicity to earthworms and soil microorganisms, with concentrations as low as 0.02 mg/kg significantly reducing earthworm populations [41]. Although CL-20 does not directly affect the growth of higher plants like alfalfa and perennial ryegrass, it can bioaccumulate in plant tissues, raising concerns about potential biomagnification through the food chain [42]. Additionally, studies have shown that CL-20 affects the bioluminescence of marine bacteria (*Vibrio fischeri*), the cell density of freshwater green algae (*Euglena gracilis*), and soil enzyme activity, suggesting its potential for significant ecological toxicity as it enters the water cycle [43].

### 3.2. TNT

TNT enters the human body primarily through ingestion, drinking water, and inhalation, posing serious health risks. Acute and chronic exposure to TNT and its metabolites can result in metabolic imbalances and chronic illnesses, including skin irritation, toxic hepatitis, hepatomegaly, splenomegaly, aplastic anemia, cataracts, cancer, anemia, and liver necrosis [44]. The median lethal dose (LD_50_) of TNT in rats is approximately 700 mg/kg, while in rabbits it is 500 mg/kg. Environmental exposure to TNT concentrations exceeding 2 mg/m^3^ can cause physiological discomfort and hematological alterations in humans, with an estimated LD_50_ of 1~2 g [45]. TNT exposure has also been linked to dermatitis, subacute poisoning, and chronic poisoning, manifesting as red papules, toxic cataracts, toxic gastritis, toxic hepatitis, hematopoietic system disorders, and reproductive toxicity in males [45]. Even at low concentrations, TNT exhibits toxic and mutagenic effects in humans [46]. Inhalation of high concentrations of TNT over a short period can induce cyanosis, chest tightness, and respiratory distress associated with methemoglobinemia [47]. Studies on Wistar female rats have shown that prolonged TNT exposure disrupts pituitary gonadotropin secretion, elevates serum estradiol levels, depletes the primordial follicle pool, and causes hormonal imbalances [48]. Consequently, long-term TNT exposure increases the risk of anemia and liver dysfunction in humans. Animal studies indicate that TNT adversely affects blood, liver, spleen, and other immune-related organs and may induce genetic mutations leading to genotoxicity [49]. The U.S. Environmental Protection Agency (EPA) has classified TNT as a Group C carcinogen [50].

Due to its extensive military and industrial applications, global TNT production reaches thousands of tons per month. Its testing, use, and improper disposal have led to widespread soil contamination. High TNT concentrations in soil exert acute, subacute, and reproductive toxicity on surrounding vegetation, invertebrates, and microorganisms [51]. Additionally, TNT metabolites contribute to environmental toxicity, exhibiting similar or even greater hazards than TNT itself [52]. Given its low soil affinity, TNT readily migrates and undergoes biodegradation by fungi and bacteria under aerobic and anaerobic conditions in soil and water. However, TNT and its degradation products can enter groundwater through soil runoff and eventually contaminate marine ecosystems [53]. Studies in the Baltic Sea have detected TNT and its degradation products in 98% of marine organisms, including plankton, macroalgae, mollusks, echinoderms, polychaetes, sea anemones, crustaceans, and commercially important fish species [54]. TNT and its metabolites have sublethal and chronic effects on aquatic organisms, particularly benthic species, and can lethally affect the development of fish eggs, fry, and juvenile fish [55]. Additionally, TNT exposure has been found to alter the skin microbiome of tadpoles, potentially impacting host health and immune function [56]. Beyond its environmental impact, TNT may bind to soil organic matter, entering the food chain and posing long-term risks to human health.

### 3.3. RDX

RDX enters the human body through dermal absorption, inhalation, and ingestion, causing hepatotoxicity, anemia, seizures, syncope, and, in severe cases, death [57]. Toxicokinetic studies indicate that RDX is absorbed through the intestinal mucosa following accidental oral ingestion, with peak absorption occurring within the first three hours [58]. After this initial phase, the absorption rate decreases significantly, reaching its highest blood concentration approximately 72 h post-exposure [59]. Similar findings have been observed in oral toxicity studies with young pigs, where RDX absorption peaked within three hours [60]. RDX exhibits pronounced neurotoxicity, with exposure leading to convulsions, dizziness, vomiting, and coma in humans. Symptoms typically subside within days or weeks following the removal of the exposure source [61]. The U.S. EPA classifies RDX as a Group C carcinogen, indicating its potential carcinogenic risk upon prolonged or high-dose exposure [62].

RDX is highly persistent in the environment due to its limited solubility and resistance to biodegradation, leading to long-term soil contamination and ecological risks [63]. RDX negatively impacts soil quality, inhibiting seed germination, root development, and plant growth. It also disrupts microbial communities, leading to long-term soil degradation [64]. In aquatic environments, RDX exhibits significant toxicity to freshwater fish, invertebrates, and birds. Toxicity studies have demonstrated adverse effects on fish species such as *Pimephales promelas*, *Ictalurus punctatus*, and *Danio rerio* [65,66,67]. Red-backed salamanders (*Plethodon cinereus*) exposed to RDX for 28 days exhibited neuromuscular dysfunction, including lethargy, hyper-responsiveness, tremors, and significant weight loss [68]. Bobwhite quails (*Colinus virginianus*) displayed a dose-dependent decline in feeding, weight, and egg production following RDX ingestion, with high doses proving fatal [69]. Additionally, exposure to high RDX concentrations in Caenorhabditis elegans led to germ cell apoptosis, reduced reproductive output, and clear evidence of reproductive toxicity [70].

### 3.4. HMX

The impact of HMX exposure on different species varies significantly, with notable differences in acute oral toxicity across animal species. The acute oral toxicity (LD_50_) of HMX for rodents (rats and mice) exceeds 2000 mg/kg. For birds, such as the Northern Bobwhite (*Colinus virginianus*), the LD_50_ can reach 10,000 mg/kg. However, for New Zealand rabbits, the LD_50_ is significantly lower, at 250 mg/kg for males and just 50 mg/kg for females [63].

The gastrointestinal absorption capacity plays a crucial role in determining HMX’s acute oral toxicity. Intestinal retention time is one of the most reliable predictors of HMX toxicity and species-specific sensitivity. Additionally, the neurotoxicity of HMX is a critical concern. Exposure to 200 mg/kg of HMX can cause significant damage to the central nervous system, kidneys, and liver in mice and rats [71].

In humans, HMX can enter the body through inhalation, skin contact, or ingestion. Exposure to HMX may lead to acute poisoning, characterized by seizures, nausea, convulsions, and loss of consciousness [72]. HMX accumulates in vital organs such as the heart, kidneys, liver, and brain. It exhibits mutagenic and carcinogenic properties and is classified as a Class D carcinogen, with notable neurotoxic effects [73]. Epidemiological studies suggest that HMX exposure negatively affects neurobehavioral functions in workers, increasing levels of tension, depression, anger, and confusion. Additionally, exposed workers experience declines in attention, memory, reaction speed, and manual dexterity [74].

Due to its persistence and non-degradability, HMX can leak into surrounding water and soil during production, storage, and application, leading to environmental accumulation and potential harm to organisms and ecosystems [75]. While HMX is not lethal to higher plants and earthworms, its presence reduces biomass and fertility, impairing the reproductive functions of earthworms [76]. Even at low concentrations, HMX significantly decreases earthworm reproduction, particularly affecting cocoon and larval production, indicating strong reproductive toxicity [77]. Furthermore, HMX exhibits a synergistic toxic effect with TNT and RDX in earthworms. The frequent co-occurrence of these explosives in the environment greatly amplifies the toxic impact of HMX on earthworm populations [78].

## 4. Toxicity Mechanism of Common Components in CL-20 Cocrystal Materials

Energetic materials exert complex toxic effects on biological systems, making it challenging to establish direct structure–effect relationships. This complexity presents difficulties in the application of energetic materials, the diagnosis and treatment of related health conditions, the protection of personnel exposed to these substances, and the development of new energetic compounds. Therefore, a deep understanding of the toxicity mechanisms of energetic materials is a crucial and practically significant research goal. Currently, studies on the toxicity mechanisms of energetic materials remain fragmented and are primarily linked to their most prominent toxic effects. This article will review the toxicity mechanisms of key components in CL-20 cocrystal materials accordingly.

### 4.1. The Toxic Mechanism of Action of CL-20

#### 4.1.1. Neurotoxicity

Toxic damage to the nervous system occurs through three fundamental mechanisms: direct destruction and death of neurons and neuroglial cells, disruption of electrical transmission, and interference with chemical neurotransmission [79]. Earthworms, commonly used as model organisms for studying the neurotoxicity of energetic materials, possess muscle cells with dual innervation. One pathway provides excitatory (depolarizing) signals mediated by acetylcholine, while the other provides inhibitory (hyperpolarizing) signals mediated by gamma-aminobutyric acid (GABA) [80].

The neurotoxicity of TNT in earthworms arises from secondary effects triggered by oxidative stress. In contrast, CL-20 directly targets the neurotransmission system, inducing neurotoxicity along with cascading effects such as oxidative stress via ferritin [40]. Synaptic dysfunction at the neuromuscular junction (NMJ) is believed to be the primary cause of CL-20-induced neurotoxicity, although potential disruptions in synaptic communication between the central nervous system (CNS) and peripheral nervous system (PNS) ganglia cannot be ruled out [81].

**Figure 3:**
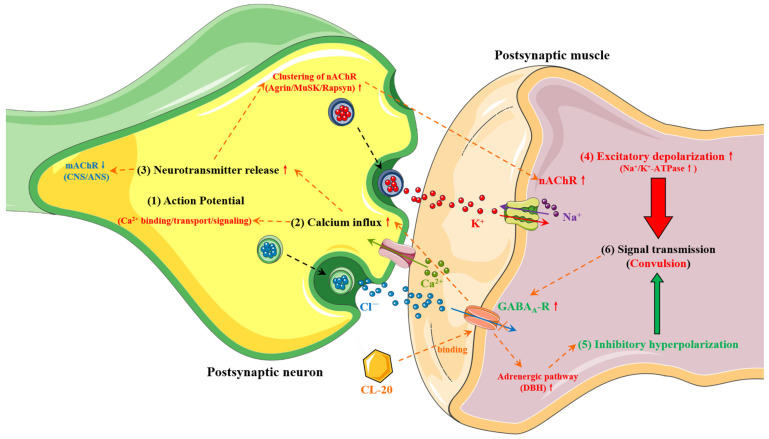
The mode of action of CL-20 on the reversible neurotoxicity induced at the earthworm double innervated neuromuscular junction (NMJ). The numbered events (1) to (6) are the basic sequential molecular events that occur at the NMJ when neuronal impulses (action potentials) reach the presynaptic neuron terminal. Abbreviations: ACh = Acetylcholine; GABA = Gamma-aminobutyric acid; nAChR = Nicotinic acetylcholine receptor; GABA_A_-R = GABA_A_ receptor; MuSK = Muscle-specific kinase; DBH = Dopamine beta-hydroxylase.

Studies indicate that exposure to CL-20 leads to reduced levels of muscarinic acetylcholine receptors (mAChRs) and decreased conduction velocity in the medial giant fiber (MGF) of *Eisenia fetida* earthworms, resulting in neurotoxic effects. However, these effects are reversible once CL-20 exposure ceases [39]. Notably, CL-20 does not affect the expression of the acetylcholinesterase (AChE) coding gene, suggesting that its neurotoxicity is not mediated through AChE inhibition but rather through interactions within the cholinergic system [82]. Research by Gong et al. further supports this, demonstrating altered expression of GABA_A_ receptor (GABA_A_-R) transcripts and modifications in cholinergic synapses [40]. Their findings indicate that CL-20 non-competitively blocks ligand-gated GABA_A_-R ion channels, resulting in changes in gene expression related to GABAergic, cholinergic, and agrin–muscle-specific tyrosine kinase (Agrin-MuSK) signaling pathways, ultimately leading to neurotoxicity (Figure 3).

CL-20 may bind to the convulsive site of the GABA_A_ ion channel, which may result in a reduction in the average duration of channel openings and a decrease in Cl^−^ channel permeability, subsequently leading to diminished inhibitory postsynaptic currents [40]. The decrease in Cl^−^ permeability can facilitate action potentials via multiple voltage-gated Ca^2+^ channels, thereby promoting intracellular Ca^2+^ influx. This process leads to an augmented excitatory depolarization as part of a feedback loop mechanism, encompassing the subsequent molecular events: (1) An augmented release of the neurotransmitter acetylcholine; (2) the aggregation of nicotinic acetylcholine receptors (nAChR) resulting in the upregulation of the Agrin-MuSK signaling pathway; (3) the stimulation of nAChR synthesis and membrane depolarization through the enhancement of Na^+^/K^+^-ATPase activity and Na^+^ influx; (4) a downregulation of muscarinic acetylcholine receptors (mAChR) within the CNS/ANS; (5) alterations in the expression of genes associated with calcium (Ca^2+^) binding, transport, and signaling pathways; and (6) an upregulation of GABA_A_ receptor expression [40]. Upon the transfer of exposed earthworms to a non-contaminated environment, the degradation of CL-20 within the organism may occur through several mechanisms: the autophagic degradation of defective CL-20-bound GABA_A_-R, which are subsequently transported to autophagosomes; the dissociation of CL-20 from its binding sites; and the metabolic processing of dissociated CL-20 by cytochrome P450 or P450-like enzymes [40]. As a consequence of these and potentially numerous additional mechanisms, CL-20 is released from the NMJ, allowing effective neurotransmission at synapses to revert to baseline levels prior to exposure. This process ultimately facilitates the recovery or reversibility of neurotoxicity induced by CL-20 [40]. Nonetheless, the intricate interrelationship among these events necessitates additional comprehensive investigation.

#### 4.1.2. Genotoxicity

The main mechanisms underlying genotoxicity encompass DNA damage, oxidative stress, protein unfolding, and the activation of p53, among other factors [83]. CL-20 has the potential to elevate levels of reactive oxygen species (ROS) and malondialdehyde (MDA) in Chinese hamster V79 cells, resulting in oxidative DNA damage and strand breaks via the oxidative stress pathway. This phenomenon is associated with alterations in mitochondrial DNA, specifically involving cytochrome C oxidase III (Cox3) and Nicotinamide adenine dinucleotide dehydrogenase subunit 1 (Nd1). The mitigation of oxidative stress has the potential to reduce the genotoxic effects associated with CL-20 [36].

### 4.2. The Mechanism of Toxic Action of TNT

#### 4.2.1. Oxidative Stress and Covalent Addition Reactions

At the molecular level, the mechanisms underlying the toxicity and carcinogenicity of TNT and its derivatives can be primarily categorized into two aspects: the oxidative stress response and the covalent binding of TNT metabolites to DNA and proteins [45]. Upon cellular entry, TNT is subjected to a sequence of enzymatic catalysis, leading to redox reactions, and consequently, it is reduced [84]. The single-electron reduction in nitro groups has the potential to significantly facilitate the generation of ROS [85]. ROS exhibit a bifunctional role within cellular environments. Under physiological conditions, ROS serve as intracellular redox signaling entities, contributing to the mechanisms of signal transduction. However, an accumulation of ROS can lead to oxidative modifications of intracellular biomacromolecules, which in turn impacts their structural integrity and functional capabilities. This excess can diminish the efficacy of antioxidant enzyme defense systems and reduce the levels of non-enzymatic proteins, such as glutathione (GSH). Consequently, the overall antioxidant defense system is compromised, impairing its ability to neutralize surplus free radicals. This disruption in homeostasis can precipitate oxidative stress, resulting in cellular damage or apoptosis [86]. Oxidative stress has been demonstrated to have a significant correlation with a range of human pathologies, including mitochondrial dysfunction, cancer, inflammation, neurological and neurodegenerative diseases, diabetes, chronic kidney diseases, aging, and DNA damage [87]. The mechanisms encompass the activation of death receptor pathways, which include death molecules and death receptors, tumor necrosis factor along with its corresponding receptors, and tumor necrosis factor-related apoptosis-inducing ligands. Additionally, the activation of mitochondrial apoptosis pathways is characterized by an increase in mitochondrial membrane permeability, the opening of permeability transition channels, and the subsequent release of cytochrome C and apoptosis-inducing factors. Furthermore, apoptosis pathways mediated by stress-activated protein kinases associated with the mitogen-activated protein kinase superfamily are also activated [88].

**Figure 4:**
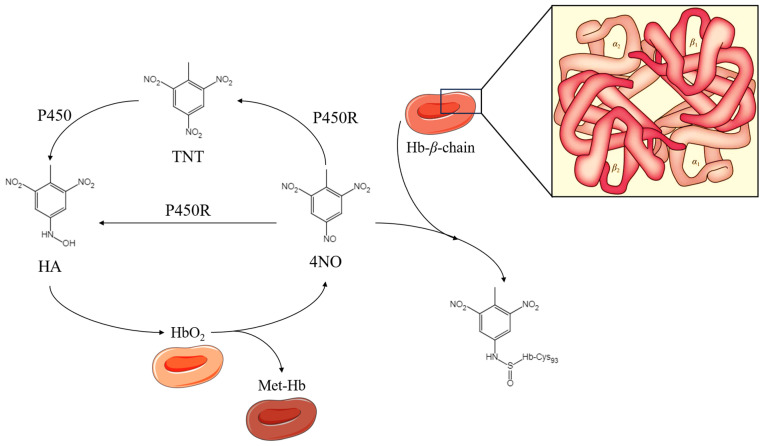
The reaction pathway of TNT and its metabolites forming adducts with hemoglobin. P450R: Cytochrome P450 reductase; Hb: Hemoglobin; HbO_2_: Oxyhemoglobin; MetHb: Methemoglobin.

In the context of the bi-electronic reduction process, nitro groups undergo metabolic conversion to yield nitroso and/or hydroxylamine groups. These metabolites possess the capacity to interact with biomolecules such as proteins and DNA, thereby contributing to toxicity and mutagenicity [89]. For instance, hemoglobin is composed of four subunits (*α*1, *β*1, *α*2, and *β*2), which encompass six reducible cysteine (Cys) residues. Notably, two *β*Cys_93_ residues are situated on the surface of the protein, rendering them susceptible to electrophilic addition reactions [90,91]. Upon entering the circulatory system, TNT is metabolically transformed into nitroso metabolites through the action of bi-electronic reductases. Subsequently, these nitroso metabolites undergo an additional bi-electronic reduction, resulting in the formation of hydroxylamine metabolites (Figure 4) [45]. The primary metabolites implicated in the blood toxicity effects of TNT are nitroso and hydroxylamine derivatives. The nitroso products will engage in oxidative modifications of the thiol groups located at the exposed termini of the hemoglobin *β*Cys_93_ residues, resulting in the formation of sulfonamide adducts. This process disrupts the natural conformation of the protein molecule, indicating that the formation of these adducts induces alterations in the secondary structure of hemoglobin, ultimately leading to a diminished capacity for oxygen transport. Furthermore, hydroxylamine metabolites have the capacity to engage in direct reactions with reduced hemoglobin, resulting in the formation of methemoglobin and nitroso metabolites. However, the molecular reaction mechanism underlying this process is not yet clear [92].

#### 4.2.2. Mitochondrial Dysfunction

Mitochondria in organisms are recognized as essential contributors to electron transport, alterations in membrane potential, ion transport mechanisms, and the transduction of apoptosis signals [93]. Initial research has demonstrated that nicotinamide adenine dinucleotide phosphate (NADPH), recognized as a crucial proton donor in the process of mitochondrial energy conversion, plays a significant role in the reduction and transformation of TNT [94]. TNT triggers cellular apoptosis via reactive oxygen species-mediated mitochondrial dysfunction. In the nematode Caenorhabditis elegans, TNT exerts an inhibitory effect on mitochondrial oxidative phosphorylation, resulting in mitochondrial toxicity and the subsequent activation of the stress response system [46]. The downregulation of genes linked to the inner membrane complex impedes electron transport within mitochondria, while impairment of the electron transport chain (ETC) leads to an overproduction of ROS. This disturbance affects the equilibrium of the antioxidant enzyme system and prompts the upregulation of gene expression associated with autophagy, thereby activating the mitochondrial unfolded protein response (mt UPR). The mt UPR results in the downregulation of genes associated with mitochondrial stress, thereby inducing dysfunction within the mitochondria [46]. Mitochondria serve as the primary organelle responsible for the toxicity of TNT in plant systems. Monodehydroascorbate reductase 6 (MDHAR6) has been identified as the primary contributor to the phytotoxic effects of TNT in Arabidopsis thaliana [95]. MDHAR6 is known to target both mitochondria and plastids, facilitating the reduction in TNT to nitro groups while concurrently oxidizing Nicotinamide adenine dinucleotide (NADH). Free radicals undergo spontaneous oxidation to revert to TNT, resulting in the production of superoxide radicals. This process perpetuates a cycle that leads to the depletion of NADH while simultaneously generating superoxide [51]. The presence of superoxides, in conjunction with subsequent hydrogen peroxide and various free radicals, engages in reactions with cellular constituents, including mitochondria, DNA, and cell membranes, resulting in cellular damage.

#### 4.2.3. The Transmembrane Transport Mechanism of TNT

The detrimental effects of TNT are associated with its adsorption, distribution, metabolism, and excretion (ADME) processes within biological organisms. The oxidative stress induced by TNT, along with its covalent interactions with DNA and proteins, is linked to its metabolites. Investigating the transmembrane transport mechanisms of TNT is essential for enhancing our comprehension of ADME processes in biological systems. In physiological contexts, the concentration of pharmaceuticals and toxic substances significantly influences their partitioning and permeation across membranes [96]. The accumulation of TNT and its metabolites results in enhanced membrane permeability [97]. The absorption of TNT molecules by the membrane does not lead to substantial alterations in the structural integrity of the membrane; however, it exerts a pronounced influence on the membrane’s permeability and various other properties [89]. The transmembrane transport of TNT molecules demonstrates a synergistic effect, whereby TNT molecules that penetrate the membrane facilitate the subsequent adsorption, accumulation, and permeation of additional TNT molecules within the membrane structure. Moreover, the interactions between solute molecules, especially those characterized by van der Waals forces, are crucial to this process [89].

#### 4.2.4. Endoplasmic Reticulum Stress and Cellular Apoptosis Pathways

The endoplasmic reticulum stress (ERS) is a critical factor in the pathogenesis of numerous diseases, including cancer, liver fibrosis, acute kidney injury, and neurogenic hearing loss [98,99,100,101]. Hepatocytes, characterized by their intricately developed endoplasmic reticulum, represent one of the most sensitive cellular types to endoplasmic reticulum stress [101]. Moreover, research indicates that oxidative stress and endoplasmic reticulum stress can mutually activate one another, thereby intensifying cellular apoptosis [102]. The calcium pumps located within the endoplasmic reticulum (ER) exhibit a susceptibility to oxidative damage. The presence of TNT induces nitroreduction and methylation oxidation processes within the liver, with a notable impact on the smooth endoplasmic reticulum [92]. The process through which endoplasmic reticulum stress leads to programmed cell death is facilitated by CCAAT/enhancer-binding protein (C/EBP) homologous protein (CHOP) and cysteine aspartate protease-4 (Caspase-4) [103]. Nevertheless, the current understanding remains ambiguous regarding whether the activation of the endoplasmic reticulum stress response constitutes a direct indication of toxicity or serves as a protective mechanism employed by cells in response to toxic agents. In order to establish a robust foundation for the treatment of individuals who have been exposed to toxic substances, it is imperative that additional research is conducted to elucidate this relationship.

Additionally, Ni et al. conducted an investigation into the toxicological impacts of TNT on various biological parameters, including lifespan, reproductive output, uterine egg counts, oocyte quantities, and the occurrence of germ cell apoptosis in Caenorhabditis elegans [52]. The findings indicate that TNT is associated with a decrease in lifespan, brood size, uterine oocytes, and egg counts in the worms, concurrently resulting in an increase in germ cell apoptosis and demonstrating transgenerational toxicity. The apoptosis of germ cells induced by TNT is governed by the fundamental apoptotic pathway alongside conserved genes that respond to genetic toxicity. Furthermore, TNT experiences swift transformation upon exposure, with the principal metabolite 4-amino-2,6-dinitrotoluene (4-ADNT) identified exclusively in both the parent and offspring generations. Nevertheless, the observed monotonic decrease in the bioaccumulation of 4-ADNT across generations of C. elegans indicates that biotransformation and bioaccumulation do not serve as the primary mechanisms underlying transgenerational toxicity. Ni et al. proposed that the detrimental effects of TNT on progeny may be associated with harm to germ cells and the reproductive system; however, additional validation is necessary.

### 4.3. The Toxic Mechanism of Action of RDX

#### 4.3.1. Neurotoxicity

RDX possesses the capability to traverse the blood–brain barrier, resulting in modifications to the expression of various genes within the brain, which subsequently leads to neurotoxic effects. In the course of examining the neurotoxic mechanisms associated with RDX across various species, including rats (*Sprague dawley*), Northern Bobwhite quail (*Colinus virginianus*), fathead minnow (*Pimephales promelas*), earthworms (*Eisenia fetida*), and corals (*Acropora formosa*), it was observed that the response to RDX exposure exhibits a significant degree of conservation among closely related species, even extending to those that lack organized neuronal systems [104].

**Figure 5:**
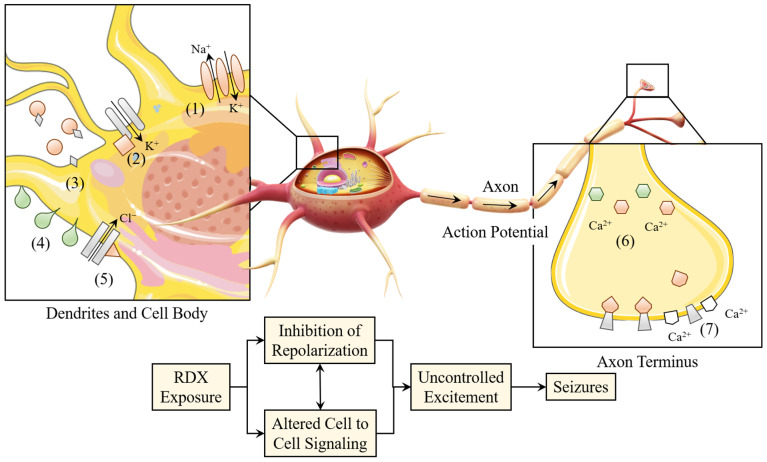
The toxic mechanism of action of RDX. Neurotoxicogenomic studies on RDX-induced epileptic seizures in northern white mice have shown significant differential expression of transcripts involved in mediating neuronal electrophysiology and signal transduction pathways (red = upregulated, green = downregulated). The overall effect of these effects is hypothesized to inhibit the repolarization potential of neuronal cells after action potentials (illustration), leading to increased neuronal excitability, epileptic seizures, and ultimately death. The affected transcripts include: (1) Na^+^, K^+^-ATPase upregulation, actively restoring neuronal repolarization; (2) Ornithine decarboxylase antizyme upregulation, regulating inward rectifier K^+^ channels affecting neuronal excitability; (3) aspartate aminotransferase gene (AspAT) upregulation, involved in buffering free glutamate (an excitatory neurotransmitter) concentration; (4) Visinin-Like Protein 1 (VSNL1) downregulation, regulating *α*4*β*2 acetylcholine receptors, increasing membrane surface expression levels and agonist sensitivity in response to intracellular Ca^2+^ levels; (5) Heat Shock Protein 70/72 (HSP70/72) upregulation, observed to promote channel opening and repolarization in association with voltage-dependent ClC-2 Cl^−^ channels; (6) differential expression of Calmodulin 2 (CALM2), which sequesters free Ca^2+^ as a neurotransmitter and neurotransmitter release factor; and (7) synaptosome-Associated Protein 25 (SNAP-25) upregulation, SNAP-25 being an important component of neurotransmitter release. The impact on these transcripts indicates that RDX has a direct effect on neuronal physiology and/or compensatory physiology, attempting to restore homeostasis within the neuronal tissue.

The elevation of calcium ions plays a pivotal role in the neurotoxic effects associated with RDX. The influence of RDX on neuronal calcium signaling is characterized by a transient elevation of intracellular Ca^2+^ concentrations, resulting in membrane excitation and subsequent neurotransmitter secretion [105]. Excitatory neurotransmitters and the rapid alterations in the electrical properties of the central nervous system, which are mediated by Ca^2+^, exhibit a significant correlation with the occurrence of epileptic seizures [106]. Nonetheless, the inhibition of Ca^2+^ channels does not entirely eliminate the elevation of Ca^2+^ levels triggered by RDX, a phenomenon that may be attributed to various underlying mechanisms [60]. Gust et al. identified a minimum of three mechanisms and various pathways linked to the neurotoxic effects of RDX [107]. The mechanisms identified encompass the diminishment of K^+^ transport across neuronal membranes, the suppression of neuronal ATPases, specifically Na^+^, K^+^-ATPase, and Ca^2+^-ATPase, as well as the inhibition of calmodulin’s regulatory influence on Ca^2+^ at axon nodes, which subsequently impacts neurotransmitter release (Figure 5). The mechanisms in question impede neuronal repolarization, augment overall neuronal excitability, and consequently result in the manifestation of epileptic seizures.

Furthermore, RDX can bind to the picrotoxin convulsive site in the GABA_A_-R chloride channel, reducing GABAergic inhibitory transmission in the amygdala, leading to reversible neurotoxicity and triggering epileptic seizures [108]. The *α*1*β*2*γ*2 subunit is estimated to constitute approximately 60% of GABA_A_ receptors and exhibits high expression levels in the cortex, thalamus, globus pallidus, and hippocampus, thereby rendering it a significant target for RDX [109]. Pressly et al. provided evidence that RDX interacts with the non-competitive antagonist (NCA) site within the GABA_A_ receptor, with a specific affinity for the threonine ring [110]. Furthermore, RDX has the capacity to enhance the upregulation of glutamate while simultaneously diminishing the downregulation of various neurotransmissions. This modulation leads to a reduction in both the frequency and amplitude of spontaneous GABA_A_-R-mediated inhibitory currents, as well as a decrease in the amplitude of GABA-induced postsynaptic currents, ultimately contributing to an increase in neuronal excitability. Nonetheless, it remains to be elucidated whether this phenomenon is attributable to overarching stress effects or the specific action of RDX [111].

Furthermore, hypoxia represents one of the mechanisms through which RDX induces neurotoxicity [112]. Upon entering the bloodstream, RDX undergoes oxidation of hemoglobin (Hb), resulting in the formation of methemoglobin (MetHb). This alteration diminishes the oxygen-carrying capacity of hemoglobin and inhibits the release of normal oxyhemoglobin, consequently leading to tissue hypoxia. The central nervous system exhibits heightened sensitivity to hypoxia, which subsequently triggers a range of clinical manifestations. Moreover, the gonadotropin-releasing hormone (GnRH) signaling pathway, along with glycolysis and gluconeogenesis, may exert indirect influences on the neurotoxicity associated with RDX [113,114]. Furthermore, RDX has the potential to induce substantial alterations in miRNA expression levels, which may result in neurotoxicity; however, the precise signaling pathways require additional comprehensive investigation [115]. Alongside the implications for the nervous system, notable pathways associated with energy and metabolic functions exhibit a shared influence. As evolutionary distance increases, the effects of neurotoxicity-related occurrences, including molecular initiation and GABA_A_-R interaction, diminish, while the broader implications for metabolic function become more pronounced [104]. Certain reports suggest that RDX may lead to increased salivation and tearing; however, the investigation conducted by Williams et al. demonstrates that RDX does not induce sialorrhea or inhibit acetylcholinesterase activity [108].

#### 4.3.2. Reproductive Toxicity

RDX stimulates the generation of ROS and triggers apoptotic pathways, while concurrently diminishing oocyte production in the model organism *Caenorhabditis elegans*. Mutant strains provide confirmation that the apoptotic process in the nematode germline is intricately linked to the fundamental apoptotic pathway as well as the signaling pathways associated with DNA damage [69]. In the gain-of-function mutant *ced-9* (n1950), there is a notable increase in the number of apoptotic cells. Conversely, in the loss-of-function mutant *ced-4* (n1162), the count of apoptotic germline cells is significantly diminished when compared to the control group. However, subsequent regulatory measures do not yield any significant alterations, suggesting that RDX exposure triggers the fundamental apoptotic pathway in the nematode [69]. In the loss-of-function mutants *cep-1* (w40), *egl-1* (n487), and *hus-1* (op241), there is no notable alteration in the quantity of apoptotic germline cells, indicating that DNA damage is a critical factor in the apoptosis process [69]. Furthermore, the outcomes of RNA sequencing indicate that the possible metabolic pathways of RDX in nematodes are associated with the functions of cytochrome P450 and glutathione S-transferase GST; however, the precise mechanisms warrant additional investigation [69]. The genes *met-2*, *set-2*, and *spr-5* are implicated in the regulation of histone methylation. Their observed downregulation in progeny suggests a correlation between the transgenerational reproductive toxicity of RDX and epigenetic changes [69].

### 4.4. The Mechanism of Toxic Action of HMX

HMX has the capacity to elicit toxic effects through the induction of oxidative stress in Chinese hamster lung fibroblast CHL and V79 cells [88,116]. 8-OHdG is generated through the attachment of a hydroxyl group to the eighth carbon of guanine, serving as a significant biomarker for oxidative damage to DNA caused by ROS and carcinogens [117]. The presence of HMX markedly elevates the cellular concentrations of 8-OHdG and ROS. GSH, recognized as a critical antioxidant within the cellular antioxidant framework, is instrumental in the scavenging of ROS and in mitigating cellular toxicity [118]. The reduction in GSH may serve as an early signaling mechanism for the initiation of apoptosis. The presence of HMX results in a reduction in cellular GSH levels, suggesting that HMX has the capacity to induce oxidative stress through the depletion of intracellular GSH [116]. In biological systems, reactive oxygen species interact with lipids, initiating peroxidation processes that yield MDA. This compound subsequently facilitates the cross-linking polymerization of biomolecules, including nucleic acids, thereby inducing detrimental effects on cellular integrity [119]. As the concentrations of HMX exposure rise, the levels of MDA exhibit a corresponding increase, suggesting that HMX has the potential to induce free-radical-mediated damage in V79 cells [116]. SOD functions as an antioxidant enzyme, playing a critical role in maintaining the equilibrium between oxidative and antioxidative processes [120]. The elevation in HMX exposure concentration results in a pattern characterized by an initial decline followed by a subsequent rise in intracellular SOD levels, suggesting a diminished capacity of cells to eliminate oxygen free radicals [116]. In conclusion, HMX has the potential to elicit cellular toxicity through the induction of oxidative stress. This process is characterized by an elevation in ROS, 8-OHdG, and MDA, alongside a reduction in GSH and SOD, resulting in DNA damage.

## 5. Speculation of the Toxicities of CL-20 Cocrystal Materials and Their Influencing Factors

While the implementation of cocrystal technology has yielded improvements in the performance of CL-20 and has augmented its efficacy in specific domains, the associated potential toxicity risk persists as a significant concern. This issue necessitates further investigation and assessment to ascertain its safety for practical applications. However, given the current absence of experimental studies regarding the toxicity of CL-20 cocrystal materials, only tentative speculation can be advanced concerning both their toxicities and the contributions of their components to their toxicities.

Utilizing the CL-20/TNT cocrystal material as a case study, the existing literature provides a foundational understanding regarding the toxicity profiles of both CL-20 and TNT. The cage-like nitramine structure of CL-20 has the potential to inflict harm on biological organisms through the release of decomposition products, including nitrogen oxides. In contrast, the toxicity associated with TNT encompasses both acute toxicity and prolonged chronic effects on various physiological systems, including the liver, blood system, nervous system, and reproductive system, in addition to its environmental toxicity. Consequently, the resultant cocrystal material is anticipated to demonstrate toxicological effects across multiple dimensions, encompassing acute toxicity, chronic toxicity, neurotoxicity, genotoxicity, reproductive toxicity, and environmental toxicity.

Nevertheless, it is not clear whether the toxicity of CL-20 cocrystals is solely due to the inherent toxicity of CL-20 and its conformers, or if new (or reduced) toxic risks arise from the interactions and/or chemical bonds formed between the components. Owing to the numerous toxicity factors and intricate interactions involved, making additional predictions proves to be challenging. Although no relevant studies exist on cocrystals of EMs, cocrystal technology has been demonstrated and employed to reduce the toxicity and side effects of various drugs, such as Theophylline, Palbociclib, 5-fluorocytosine, Oxaliplatin, etc., [121,122,123,124]. However, the molecular structures of EMs exhibit substantial distinctions from pharmaceutical molecules. For example, molecular simulations indicate that in IMX-104, DNAN and RDX may have a synergistic effect, amplifying its toxicity [125]. Due to the structural resemblance between RDX and CL-20, analogous interactions may also occur within CL-20 cocrystal materials. Overall, the complex interactions among components within EM mixtures, including superposition, synergy, or antagonism, may exert influence on their toxicological manifestations. Complex interactions may arise among the constituents of CL-20 cocrystal materials, potentially influencing the overall toxicity of the material and complicating the prediction and evaluation of its toxicities. The specific toxic effects, however, remain to be elucidated through experimental research.

In addition, CL-20 cocrystal materials may generate poisonous intermediate products during breakdown or metabolism. These intermediates may exhibit potential toxicity, carcinogenicity, and mutagenicity. For instance, studies demonstrate that TNT degradation products 2-amino-4,6-dinitrotoluene (2-ADNT) and 4-amino-2,6-dinitrotoluene (4-ADNT) exhibit combined toxicity, carcinogenicity, and mutagenicity [126]. When TNT forms cocrystal materials with CL-20, these toxic substances likely persist within the degradation products of the resulting cocrystals. Furthermore, the components of CL-20 cocrystal materials may modify their degradation paths via intermolecular interactions, thereby influencing the decomposition routes and the production of hazardous byproducts. Such alterations may result in fluctuations in the kind and volume of hazardous substances, thereby affecting the material’s toxicity profile. Research on metallic cocrystals has demonstrated exceptional selectivity in decomposition. For example, 2-chloroethyl ethyl sulfide (CEES) can be selectively photo-oxidized to 2-chloroethyl ethyl sulfoxide (CEESO) without generating highly toxic 2-chloroethyl ethyl sulfone (CEESU2) [127].

However, previous studies of Song et al. indicate that void defects in CL-20/TNT cocrystals create hot spots upon impact, which significantly accelerate the decomposition of CL-20/TNT cocrystals and promoting the nitro group detachment in CL-20 while concurrently inhibiting its polymerization and adsorption [128]. It suggests that cocrystal structures in EMs may also facilitate the rapid release of toxic degradation substances into the environment, thereby elevating risks to ecosystems and human health. Therefore, it is imperative to investigate the toxicity of eutectic materials through rigorously designed experimental studies.

In summary, the toxicity analysis of CL-20 cocrystal materials is essential for scientific integrity and is fundamental to safeguarding human health, environmental safety, and technological application. Considering the multifactorial impact on toxicities of CL-20 cocrystals, experimental assessment of their toxicities is imperative to inform their production and application. Toxicity testing constitutes an essential step in advancing CL-20 cocrystal materials from laboratory research to engineering applications, fulfilling an irreplaceable role in safeguarding the sustainable development of military equipment and environmental safety. A detailed examination of its toxicity processes will provide data support for facilitating its large-scale implementation.

## 6. Conclusions and Future Directions

Cocrystal technology may substantially reduce the sensitivity and enhance the application potential of CL-20 by integrating other energetic elements such as TNT, RDX, and HMX. The design, synthesis, and use of CL-20 cocrystal energetic materials have emerged as a prominent research focus in the area of energetic materials during the last decade. Since the first successful synthesis of CL-20/TNT cocrystal materials in 2011, researchers have produced many CL-20 cocrystal materials using diverse methodologies. These eutectic materials exhibit substantial enhancements in thermal stability, energy density, and mechanical characteristics. Furthermore, computational biology is crucial for the prediction and optimization of cocrystal materials. Despite advancements in the experimental synthesis of common crystal energy materials at the laboratory level, research concerning their toxicity effects, mechanisms, and protective measures remains largely undeveloped. This paper examines the development process of CL-20 cocrystal materials, evaluates the toxic effects of various components including CL-20, TNT, RDX, and HMX, and analyzes their toxic mechanisms. Additionally, it speculates on potential toxic factors associated with CL-20 cocrystal materials by synthesizing existing research findings and pertinent data.

CL-20 can cause lung inflammation, pulmonary edema, and multi-system damage. It exhibits mutagenic properties, weak skin sensitization, and no significant skin irritation. Additionally, CL-20 is persistent in soil, posing acute and chronic toxicity risks to earthworms and nematodes, with greater toxicity to soil microorganisms. It can accumulate in plants, leading to potential biomagnification, and affects marine bacteria, freshwater algae, and soil enzyme activities, raising concerns about extensive ecological toxicity. The neurotoxic mechanism of CL-20 is linked to GABA ion channels and acetylcholinergic factors, while its genotoxicity is closely associated with DNA damage induced by oxidative stress.

TNT affects human health through ingestion, inhalation, and dermal exposure, causing skin irritation, hepatitis, anemia, and carcinogenic effects. Animal studies indicate that TNT damages the blood, liver, and spleen and may induce genotoxicity. Its widespread use results in soil contamination and exerts acute, subacute, and reproductive toxicity on vegetation, invertebrates, and microorganisms. The environmental migration and degradation of TNT metabolites pose a threat to soil and aquatic organisms, leading to long-term harm to human health. Current research has extensively explored the toxic mechanisms of TNT, including oxidative stress, covalent binding, mitochondrial dysfunction, ER stress, and apoptosis.

RDX enters the human body through skin contact, inhalation, and ingestion, causing hepatotoxicity, anemia, seizures, syncope, and, in severe cases, death. It exhibits reversible neurotoxicity and potential carcinogenicity. RDX is resistant to decomposition and has low soil solubility, leading to long-term contamination that degrades soil quality, affects plant growth, and disrupts microbial communities. The neurotoxicity of RDX is primarily mediated through GABA receptor modulation, while its reproductive toxicity is linked to apoptotic pathway regulation.

The acute oral toxicity of HMX varies significantly across species and is strongly correlated with its absorption and retention time in the gastrointestinal tract. HMX is neurotoxic, and human exposure via inhalation, skin contact, or ingestion can lead to acute poisoning, seizures, nausea, convulsions, and loss of consciousness. It accumulates in the heart, kidneys, liver, and brain and exhibits both mutagenic and carcinogenic properties. Due to its persistence and low degradability, HMX accumulates in the environment, impacting ecosystem functions. Although not lethal to higher plants and earthworms, it reduces biomass and fertility, affecting earthworm activity and reproduction, particularly cocoon and larval production. HMX, TNT, and RDX exhibit synergistic toxicity in earthworms, further increasing ecological risks. However, the toxicological mechanisms of HMX remain poorly understood, with studies suggesting it induces oxidative stress leading to DNA damage and apoptosis.

The toxicological effects of CL-20 cocrystal materials present a complex challenge that spans chemistry, biology, and environmental sciences. Although cocrystallization technology enhances the performance of CL-20 to some extent, its potential toxicity risks require further investigation to ensure safe application. A comprehensive evaluation of CL-20 eutectic materials is essential, and future research should focus on the following aspects:

1. Acute toxicity, long-term toxicity, and ecological toxicity of cocrystal materials on different organisms, and their toxic action mechanism. Researchers should conduct comprehensive studies on acute, chronic, and ecological toxicity of cocrystal materials across different organisms. A deeper investigation into the metabolic pathways of eutectic materials in various species is essential to understanding their physiological impacts and potential disruptions to ecosystem balance.
2. The interaction of eutectic material components and its influence on the overall toxic effect of materials. The interactions between different energetic material components can significantly influence overall toxicity. Understanding these interactions is crucial for designing safer eutectic materials with minimized adverse effects.
3. Utilizing computational chemistry and molecular simulation techniques, researchers can develop QSAR models. These models provide a scientific basis for designing safer eutectic materials by predicting potential toxicity risks at the early stages of material development.

In conclusion, CL-20 cocrystal materials hold great potential in the field of energetic materials, but their toxicity requires further research to ensure safe use in both military and civilian applications. A deeper understanding of their toxic mechanisms will not only enhance safety assessments but also contribute to the development of effective toxicity mitigation strategies. With advancements in science and experimental technology, future research is expected to provide better insights into CL-20 cocrystal toxicity and enable the implementation of protective measures, ensuring its safe application in energetic materials.

## Data Availability

No new data were created or analyzed in this study. Data sharing is not applicable to this article.
